# **Rethinking Innovation in Drugs:** A Pathway to Health for All

**DOI:** 10.1017/jme.2023.157

**Published:** 2023

**Authors:** Mariana Mazzucato

**Keywords:** Drug Innovation Policy, Health Equity, Public-Private Collaboration, Intellectual Property Reform, Mission-Oriented Governance

## Abstract

This article discusses the misalignment of the drug innovation model in the US with broader societal goals. The paper calls for a reconfiguration of this model to prioritize the common good and ensure equitable access to health innovations. The article stresses the importance of adopting a mission-oriented approach to shape the drug market, including reforming intellectual property rights.

During the COVID-19 pandemic, the rapid development of vaccines stood as a testament to what can be achieved when human ingenuity and private-sector involvement receive significant public support. Yet, within a year of vaccine development, high-income countries hoarded 870 million excess doses while intellectual property rights remained in the hands of a few pharmaceutical companies.^1^ As a result, the world entered a stage of “vaccine apartheid”, as World Health Organization (WHO) Director-General Tedros Adhanom Ghebreyesus pointed out.^2^ The global excess death toll from COVID-19, which stands at 14.9 million^3^, is a stark example of what happens when we fail to harness the power of innovation to create an economy that serves the common good.^4^ While the WHO declared an end to COVID-19 as a public health emergency of international concern on 5 May 2023,^5^ the structural issues of inequitable access to health persist. Many people remain excluded from the benefits of health innovation, creating unacceptable inequities that often exacerbate existing hardship. Health innovation is futile if its rewards are not equitably shared.^6^

Governing health innovation for the common good is critical to promote broader public benefits and equitable access. Decentralizing innovation and manufacturing companies to ensure global resilience is a crucial enabler for achieving this goal. As the WHO Council on the Economics of Health for All, which I chaired, has argued, this requires a major shift from a model where innovation is seen as being driven by market forces, to a model that is collectively governed in the public interest.^7^ Rather than viewing patents solely as revenue generators or incentives for pharmaceutical companies, which then serve as wealth transfers to shareholders, patents should effectively stimulate productive entrepreneurship and ongoing innovation. To achieve this, the criteria for granting patents should be more stringent, and patents should exclusively cover truly novel and inventive areas, particularly focusing on downstream inventions to prevent the privatization of essential research tools, processes, and technology platforms. Innovation strategies should align with cross-sectoral missions to deliver health for all.^8^

The US, despite having one of the most dynamic innovation ecosystems, presents the poorest health outcomes among high-income countries.^9^ Americans face a higher likelihood of dying young from preventable causes compared to their counterparts in comparable nations.^10^ While the US outspends every other high-income country on health care, its performance on various health and healthcare metrics often falls short. The primary barrier to healthcare access is affordability. A significant number of Americans are either priced out or lack coverage due to excessive administrative complications. Additionally, high out-of-pocket expenses deter nearly half of working-age adults from seeking timely care.^11^ Central to this conundrum is the structure of the drug market.

The public sector is the main engine behind drug innovation in the US. Founded in the 1880s, the National Institutes of Health (NIH) is the country’s primary public agency responsible for biomedical and public health research. The NIH, a component of the US Department of Health and Human Services, plays a vital role in the research and development of new medicines. It has directly or indirectly contributed to the research that led to the development of more than 99% of the 356 drugs approved by the Food and Drug Administration (FDA) between 2010 and 2019. This achievement represents an approximate total investment of $1.44 billion.^12^

Governing health innovation for the common good is critical to promote broader public benefits, equitable access, and the decentralization of innovation and manufacturing companies to ensure global resilience.

The NIH plays an instrumental role in fostering synergies between academic-based innovators backed by federal government funding and innovations co-developed in both publicly funded and commercial institutions. Much of the NIH’s support for the drug industry is concentrated on discovery and the early stages of development. This includes funding high-risk projects where the private sector might be hesitant to engage. As such, the NIH’s contributions in this realm are pivotal for identifying new treatments. However, public funding also significantly impacts the later stages of drug development, including both proof and testing. Furthermore, drugs associated with late-stage public funding are more likely to receive expedited FDA approval or be designated as first-in-class, hastening their market entry and further reducing commercialization risks.^13^

The influence of the US government on the drug market is not limited to the development of new treatments. Another highly significant mechanism through which the US government supports drug innovation is public procurement. The US government is by far the largest single purchaser of prescription drugs through programs like Medicare and Medicaid. It can take measures to ensure that taxpayer funds are used to prioritize access for patients to meaningful pharmaceutical innovation.^14^

Perhaps one of the most recent and highly visible examples of public funding supporting pharmacological developments is the creation of the transformative mRNA COVID-19 vaccines. In this instance, not only did the NIH and the US government provide significant support for the pivotal discoveries and development — estimated at as much as $31.9 billion for mRNA vaccine technology from 2000 to 2020 — but they also ensured a guaranteed market for the final stages of development.^15^ For instance, Moderna received nearly $1 billion in research aid from the US government for its COVID-19 vaccine and secured a deal worth up to $1.5 billion to supply 100 million doses. In total, the company has garnered close to $2.5 billion in R&D and supply funding from the government for its vaccine program.^16^ Yet, Moderna refused to share its technology with others, including the South African mRNA Technology Transfer Hub, an initiative aimed at accelerating vaccine development in middle- and low-income countries.^17^ While Moderna did pledge to refrain from enforcing patent protections during the pandemic, excessive patenting is still a massive potential barrier to the development and distribution of treatments for other diseases, such as HIV and cancer.

The public sector is deeply involved in the development of prescription drugs and is a dominant player in the market. Between 2008 and 2017, approximately 25% of approved drugs were based on publicly funded research.^18^ In the fiscal year 2021, Medicaid spent approximately $80.6 billion on outpatient prescription drugs and, after collecting $42.5 billion in rebates, had a net drug spending of approximately $38.1 billion, which is about 10% of a market valued at $429 billion.^19^ However, it appears that the government has relinquished its ability to shape a more equitable and affordable market. Between 2008 and 2021, launch prices for new drugs increased by 20% per year.^20^ More than half of these drugs were found to have a low rate of added therapeutic benefit, accounting for $19.3 billion in estimated annual net spending. This represents 11% of the total net Medicare prescription drug spending in these years. ^21^ Of 81 top-selling drugs, only 27% were rated as having high therapeutic value by the FDA.^22^ A significant number of U.S. patients are using drugs of limited value at a substantial cost.

However, the current structure of drug markets in the US prioritizes economic profits over health, leading to the privatization of public investments and allowing non-competitive structures to shape predatory markets. To address this situation, it is imperative for the US Government to shift from reactively addressing market failures to proactively and collaboratively shaping markets that prioritize human health.^23^

First, it is essential to recognize that health innovation emerges from collective intelligence. Numerous stakeholders, encompassing public institutions, private entities, university research departments, and civil society organizations, contribute to the development of medical solutions. The architecture of drug research and development necessitates a thoughtful reform to prioritize the public interest. Such a shift entails cultivating reciprocal relationships between public and private sectors, driven by mutual objectives. The focus should shift from exploitative arrangements, where public investments flow to private sectors with minimal prerequisites, to ones that actively synchronize with societal needs. Mandating conditions for public funds directed towards health-related research and development can ensure not only affordability and equitable access but can also encourage the re-investment of returns into continued innovation in health.^24^ The Oxford-AstraZeneca COVID-19 vaccine, developed with the help of government investment, included provisions to keep prices low, limit profits during the COVID pandemic and ensure knowledge sharing for public health.^25^ During the pandemic, actions taken by the government and the company were clearly conditioned to benefit public health. This contrasts with the trend of monopoly pricing exercised by other companies which used strategic patenting to block competitors.

Second, it is imperative to bolster financial commitments to medical research and development, viewing this as a strategic long-term investment rather than a short-term expenditure, and to protect existing budgets, including that of the NIH in the US. But the quality of this finance is as important as its quantity. This means patient, long-term finance that directed towards achieving the goal of health for all, and that is governed with a view to maximizing its public benefit.^26^

Third, it is crucial to leverage procurement mechanisms to shape market opportunities that align with public health needs.^27^ Timely evaluations of collective demands can enable large-scale procurement, consequently reducing costs. Initiatives like Brazil’s Productive Development Partnerships and Mexico’s Consolidated Drug Purchase stand as exemplary models of centralized public procurement strategies.^28^ These approaches curtail expenses for public health. Adopting a comparable strategy in the US could wield a significant influence on market dynamics, helping to ensure that the public receives top-tier therapeutic drugs at competitive price points. Moreover, procurement budgets have the potential to be used strategically to maximize public value, beyond seeking the lowest cost option, for example through mission-oriented approaches that create market demand for needed products and services, or outcomes-oriented approaches that emphasize a public health outcome rather than a specific way of achieving it.^29^

Reconfiguring the US drug innovation landscape to prioritize the common good and ensure equitable access to health innovations requires a different approach to how health innovation is governed. While funding is essential, it alone is not the solution. Government should adopt a mission-oriented approach to drug innovation, setting bold goals related to public health that serve to catalyze innovation and investment — goals that prioritize improved patient outcomes, reduction in disease prevalence, and access equity.^30^ Achieving such bold goals would necessitate a reform of intellectual property rights.^31^ Moreover, it would require a shift in how collaborations between the public and private sectors are structured to recognize that innovation results from a collective effort, valuing contributions from both public and private entities. And it would require governments to foster collaboration across different ministries, thereby avoiding the compartmentalized governance of health. The excessive tendency of governments to outsource key operations has unfortunately weakened these capacities.^32^

Reflecting on the profound impacts of COVID-19 and its significant human, economic, and social costs, governments can set a different course. They can create a new end-to-end health innovation ecosystem governed for the common good — one that prioritizes human well-being over short-sighted profit gains, and not just in one country but in all regions of the world.^33^
